# Unlocking *Index Animalium*: From paper slips to bytes and bits

**DOI:** 10.3897/zookeys.550.9673

**Published:** 2016-01-07

**Authors:** Suzanne C. Pilsk, Martin R. Kalfatovic, Joel M. Richard

**Affiliations:** 1 Smithsonian Libraries, Washington DC, USA

**Keywords:** Metadata, Digitization, Linked Open Data

## Abstract

In 1996 Smithsonian Libraries (SIL) embarked on the digitization of its collections. By 1999, a full-scale digitization center was in place and rare volumes from the natural history collections, often of high illustrative value, were the focus for the first years of the program. The resulting beautiful books made available for online display were successful to a certain extent, but it soon became clear that the data locked within the texts needed to be converted to more usable and re-purposable form via digitization methods that went beyond simple page imaging and included text conversion elements. Library staff met with researchers from the taxonomic community to understand their path to the literature and identified tools (indexes and bibliographies) used to connect to the library holdings. The traditional library metadata describing the titles, which made them easily retrievable from the shelves of libraries, was not meeting the needs of the researcher looking for more detailed and granular data within the texts. The result was to identify proper print tools that could potential assist researchers in digital form. This paper outlines the project undertaken to convert Charles Davies Sherborn’s *Index Animalium* into a tool to connect researchers to the library holdings: from a print index to a database to eventually a dataset.

Smithsonian Libraries

Sherborn’s microcitation of a species name and his bibliographies help bridge the gap between taxonomist and literature holdings of libraries. In 2004, SIL received funding from the Smithsonian’s Atherton Seidell Endowment to create an online version of Sherborn’s *Index Animalium*. The initial project was to digitize the page images and re-key the data into a simple data structure. As the project evolved, a more complex database was developed which enabled quality field searching to retrieve species names and to search the bibliography. Problems with inconsistent abbreviations and styling of his bibliographies made the parsing of the data difficult. Coinciding with the development of the Biodiversity Heritage Library 
(BHL) in 2005, it became obvious there was a need to integrate the database converted *Index Animalium*, BHL’s scanned taxonomic literature, and taxonomic intelligence (the algorithmic identification of binomial, Latinate name-strings). The challenges of working with legacy taxonomic citation, computer matching algorithms, and making connections have brought us to today’s goal of making Sherborn available and linked to other datasets. Partnering with others to allow machine-to-machine communications the data is being examined for possible transformation into RDF markup and meeting the standards of Linked Open Data. SIL staff have partnered with Thomson Reuters and the Global Names Initiative to further enhance the *Index Animalium* data set. Thomson Reuters’ staff is now working on integrating the species microcitation and species name in the ION: Index to Organism Names project; Richard Pyle (The Bishop Museum) is also working on further parsing of the text. The *Index Animalium* collaborative project’s ultimate goal is to successful have researchers go seamlessly from the species name in either ION or the scanned pages of *Index Animalium* to the digitized original description in BHL - connecting taxonomic researchers to original authored species descriptions with just a click.

Biodiversity Heritage Library

: Index to Organism Names project

## Background

The Smithsonian Libraries’ collections support the varied museums and research centers that support the mandate for the “increase and diffusion of knowledge” established by the benefactor James Smithson. The diversity of the subject matter in the Libraries collection reflects the range of topics, disciplines and activities undertaken by Smithsonian researchers. The Libraries has developed along the lines of the Institution to support the vast array of topics that has become the largest complex of museums and research centers in the world with 20 libraries supporting 19 museums and 9 researcher centers. The Institution’s natural history collections date back to the 18^th^ century and have been collected to assist in the study and stewardship of the extensive specimen collections. The United States National Museum was established within the Institution in 1858 and moved into a separate, individual museum in 1910. The Natural History Library collections of Smithsonian Libraries have grown in conjunction with the National Museum to help researchers identify and document specimen collections. With a substantial amount of focus since its founding on classic collections-based research of systematics and taxonomy, the collection in the library supports the discovery of species and naming. The reliance on historical literature to perform the work has made for a strong library collection. The Smithsonian Libraries has grown to take on a role of providing authoritative information and creates innovate services for the curators, scientists, and researchers. (Smithsonian Institution Libraries. *Rare Books and Special Collections in the Smithsonian Institution Libraries*. 1995) This includes the move towards providing the necessary information in digital form alongside the traditional print collections.

To build and preserve along with the supporting of present day research needs, the Libraries looks for ways to have the information within the collection reach the needed patron whenever and wherever they may be. Acquiring digital data, electronic journals and resources and current research database subscriptions is one aspect of the Libraries reach. Scanning the holdings of collections to provide better access was another step towards the delivery of critical information to the researchers. The Libraries’ first started digitization projects in 1996. By 1999, a full-scale digitization center was in place and rare volumes from the natural history collections, often of high aesthetic value, were the focus for the first years of the program. The resulting beautiful books made available for online display were successful to a certain extent; but it soon became clear that the data locked within the texts needed to be converted to more usable and re-purposable form via digitization methods that went beyond simple page imaging. There was a critical need to include text conversion elements. This “freeing the data locked on the page” began to be the goal of the Smithsonian Libraries’ digitization program. The online version of reference sources began to be scanned with the text created into datasets and was the natural progression from the initial tomes with pleasing plates. Sherborn’s *Index Animalium* was one of the Libraries first attempts at digitization for database conversion.

## I.

Smithsonian Libraries is a traditional library with books on shelves with librarians and staff ready to assist the patron with their information requests. Traditional library description of monographs and serials is based on standards within the library and information sciences. The inventories of the holdings of large academic libraries require standardized practices and efficiencies of scale to accomplish sophisticated catalogues. The Libraries cover a wide range of topics from art to zoology and is geographically located across the United States and Panama. The data that is captured for each title assists in the physical allocation of material. Yet, the granularity of the descriptive data is effectively only at the title level and does not delve into the contents, chapter, article or page level with in each title, and does not index the specific details within the texts. Most libraries’ tools dating back to the card catalogues to the current online integrated library catalogues, have found that the metadata describing titles has worked with limited success. The discovery aspect of this overarching or “high” level of metadata limits the results of inquiries but sufficed for physical discovery of the titles.

Taxonomic research requires the specific citation of descriptions of species. The International regulatory codes for identifying and naming of species require in-depth research on species and genus. The rules are quite clear that when naming species, the name is considered fully formed once the description is published and available (International Commission on Zoological Nomenclature’s International Code, http://www.iczn.org/iczn/index.jsp; See specifically Article 8 and Article 11). Major natural history libraries and the Smithsonian’s National Museum of Natural History Library, specifically, have served the function of ensuring that publications of species names are stored and the publications are available. Yet the librarian standards of description of these materials has fallen short of the needs of the taxonomic researcher in identifying exactly where descriptions are located within these publications – the page level metadata and the data within the page is lacking.

**Figure 1.**
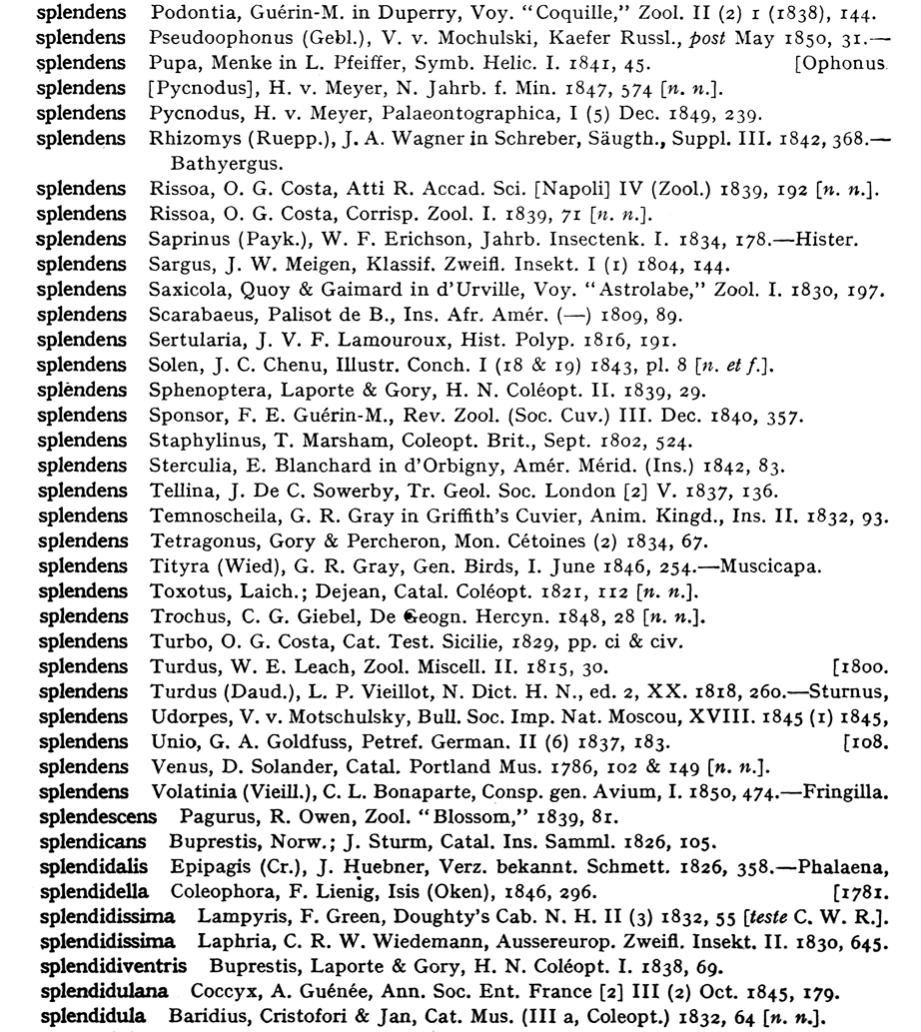
Page from *Index Animalium* showing examples of microcitations.

Smithsonian Libraries staff met with researchers from the taxonomic community to understand their path to the literature and identified resources used to connect to the library holdings. The traditional library metadata describing the titles on the shelves of libraries was not meeting the needs of the researcher looking for more detailed and granular data within the texts. Their own bibliographies and indexes were required to pinpoint the data needed. From those sources, data points had to be mapped to the library search interface (the online catalogue) with different terminology and assumptions. Each individual researcher had to interpret and translate access points to locate the desired material.

As seen in the other essays within this compilation, Charles Davies Sherborn stepped in to fill the data needs that the traditional library catalogues were not and could not meet. (Neal Evenhuis. “Sherborn: Work history and impact of bibliography, dating and zoological informatics.”) The beauty of his *Index Animalium* are the microcitations for a species giving the genus, species, author, abbreviated title of publication, and the critical date and page specifics. This level of access within the texts of monographs and serials is the data that the libraries were failing to deliver. Smithsonian Libraries saw that Sherborn’s *Index* was actually a data set that needed to be liberated off the printed page and made available digitally. The microcitations were needed in the electronic world to interact with taxonomists working in the digital world – writing, citing, and interacting with their research. The first task at hand was to scan and make available a fully searchable *Index Animalium*.

Funded by the Atherton Seidell Endowment Fund, SIL contracted to have the entire set of 30+ volumes scanned: cover to cover, over 9,000 page images. Subsequent to the imaging, the entire *Index* was re-keyed into a database. Spot checked and refined with the vendor, the final database has an accuracy rate of 99.995% and consists of over 430,000 lines of useful data. The *Index Animalium* electronic version is available at http://www.sil.si.edu/digitalcollections/indexanimalium/.

## II.

The first goal of the digital e-version of Sherborn’s Index, was to provide to the world a searchable version of the full tex of the index and the accompanying bibliographies. As the project continued, it became a mission to identify every volume that Sherborn examined in creating the Index. Once identified, the volumes could then be physically located with first preference being our own Smithsonian Libraries’ collection. If the title was not in Smithsonian’s holdings, a location would be sought within the realm of natural history libraries. This layer of access was to assist anyone using the online *Index Animalium*’s microcitations to be able to locate the book that Sherborn references.

Sherborn states in the Epilogue of *Index Animalium*, March 1922: “In any well-appointed Natural History Library there should be found every book and every edition of every book dealing in the remotest way with the subjects concerned.” With over 7,700 titles listed, Sherborn gives the most comprehensive list of all important works in the study of zoology. The four bibliographies scattered throughout the multi-volume Index records every title that Sherborn examined. He included indications if the work had no systematic zoological name, no Linnaean names, inconsistent binomial names, no specific names mentioned, or if no new species were found in the texts.

Researching all the potentially related species is required for the study of species naming. The Smithsonian Libraries’ online version of Sherborn’s *Index* is aimed to facilitate the researcher locating all the texts that are referenced. Sherborn’s bibliography, though a very comprehensive list of important titles, is not complete in the descriptions of these titles. His use of inconsistent abbreviation, “ibid” indications, use of shortened titles, and other idiosyncrasies has made identifying the exact titles challenging.

**Figure 2.**
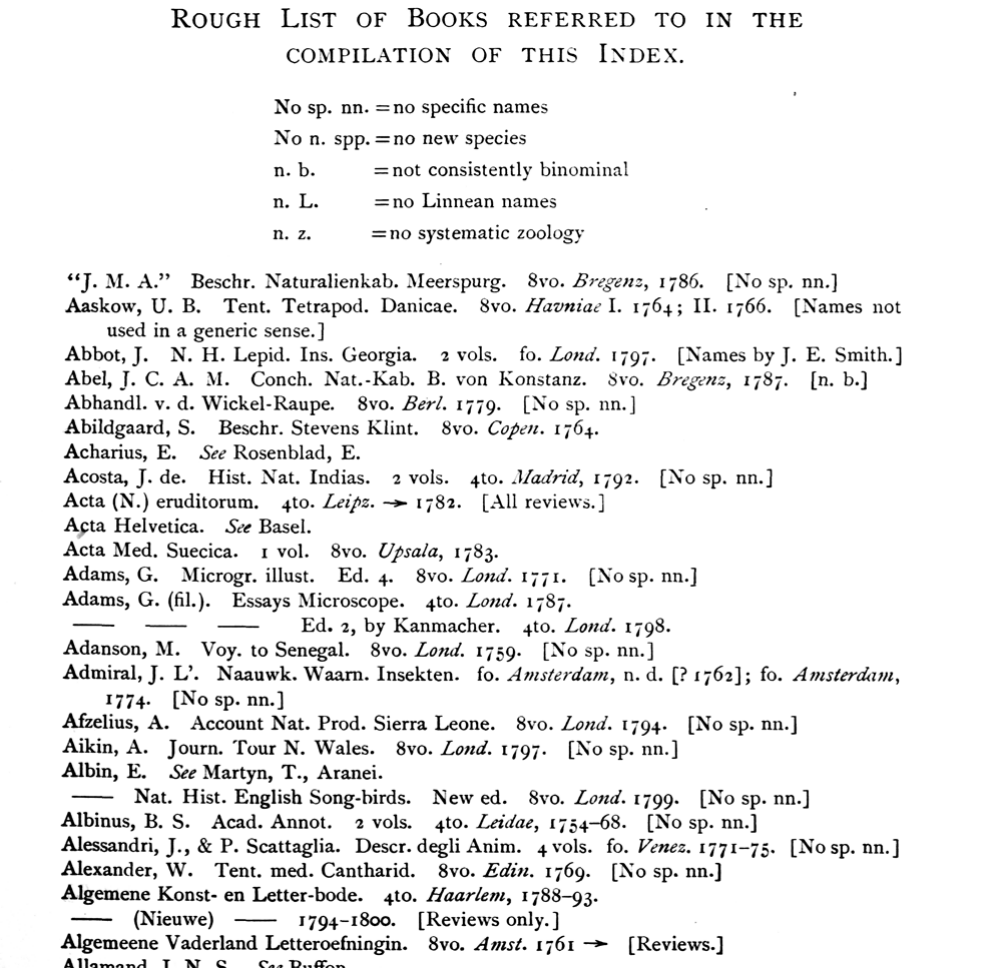
Example of the first page of the first bibliography (Sherborn. *Index Animalium*, Vol. 1, p. xi).

## III.

Smithsonian Libraries first foray into moving beyond pretty books to creating datasets faced many challenges including re-assessing the actual needs and deliverables of the project. The online project morphed from the initial basic scanning of the text – to a searchable database – to a goal of connecting each microcitation to the proper line in the bibliography – to the goal of having the microcitation connected to bibliography connected to physical location of the text. Difficulties emerge when computer-to-computer resolving of microcitations and bibliography entries were attempted. Most problematic was the use of computer scripts against Sherborn’s inconsistent notation made it impossible for clear connections of species citations to title citation to be made in a systematic way.

**Figure 3.**
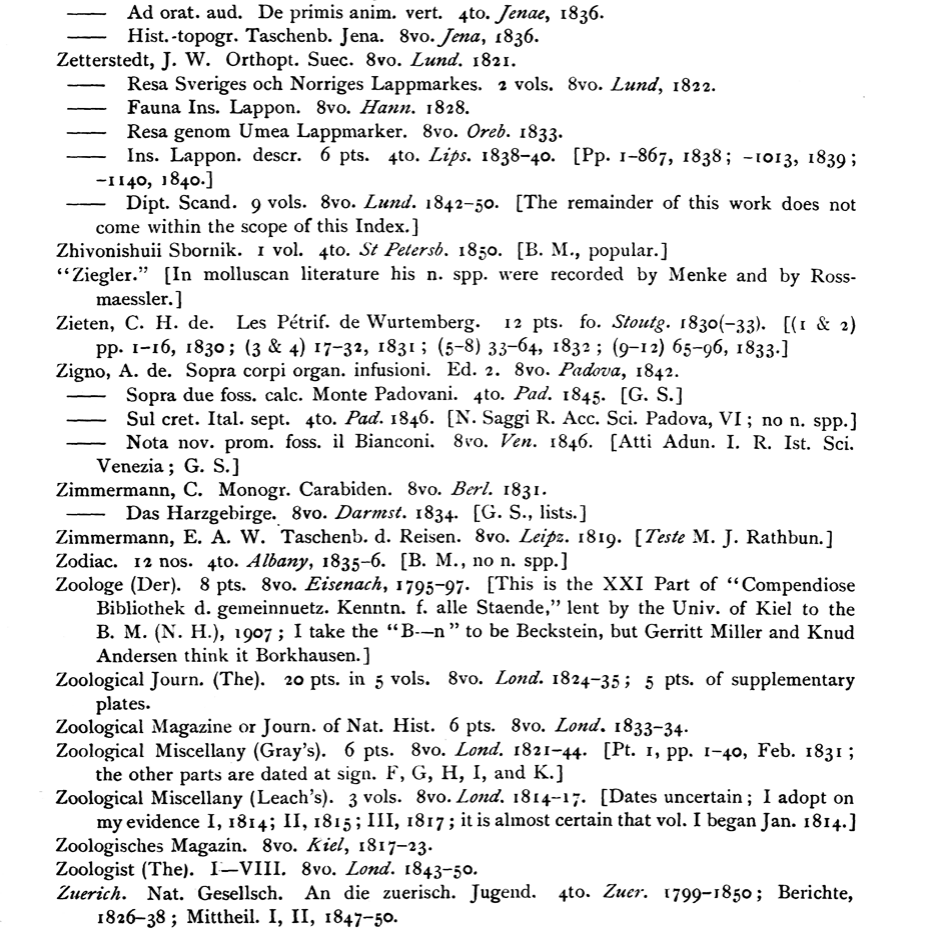
One example page from *Index Animalium* bibliography.

Simple Regular Expressions were used to break apart the re-keyed text of Sherborn based on the lessons learned by MBL WHOI Library’s project for Neave’s *Nomenclator Zoologicus*. (*Nomenclator Zoologicus* online version from uBio, Marine Biological Laboratory, Woods Hole Oceanographic Institution http://uio.mbl.edu/NomenclatorZoologicus/) Regular Expressions are a simple syntax particularly suited for identifying and dividing up textual data by looking at patterns, punctuation, and even character strings or sequences. David Remsen and Patrick Leary (formerly at MBL/WHOI) used these parsing techniques to isolate titles within the *Index’s* species citations. Using those strings they used comparisons of strings to match against the bibliography citations. Documentation regarding the parsing of *Index Animalium* data is found at http://uio.mbl.edu/Sherborne/index.html.

**Figure 4.**
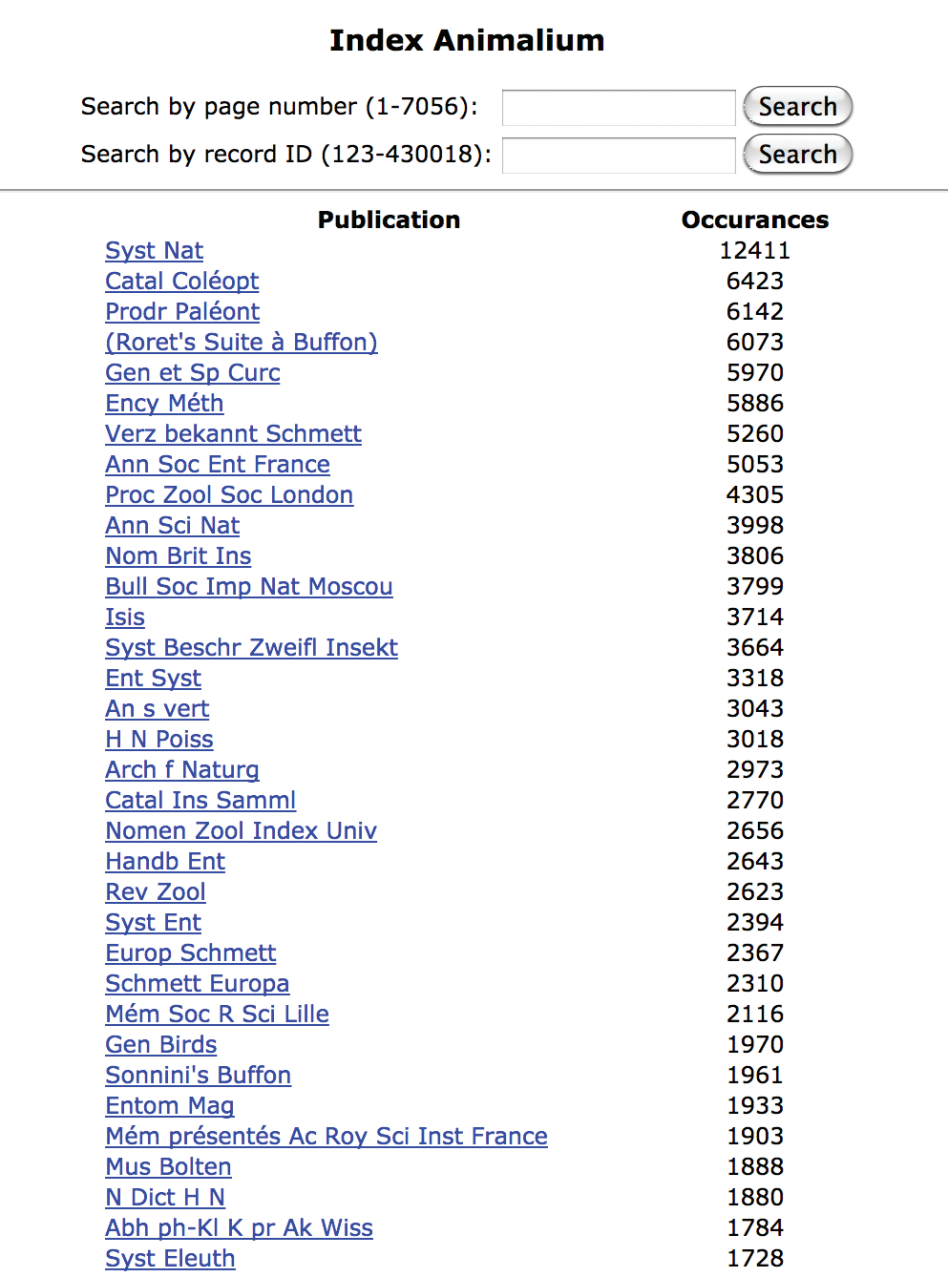
uBio parsing showing count of abbreviations found in publication area of microcitation.

As seen in some of the examples below, there were some high accuracy results at times and mixed results in others. Problems with titles that are extremely common in the field of taxonomy, that Sherborn abbreviated in a way that the researcher could recognize when reading the citations, fall short when attempting to use computerized matching. Sherborn was not consistent in his abbreviation within the microcitations. Within the bibliography, he was not consistent with title, author, editor, edition, volume, publisher, or places of publication abbreviations. He was not always consistent on what he decided to abbreviate or how he formed the abbreviations. Having a systematic string matching between the citations to the bibliography did not prove to provide the clean matching that was needed for unambiguous one to one matching. The metadata fields do not line up for easy comparison and matching; for example: author to author versus author to editor. Century-old systems of notation translated into library standard database structure have been a road-block in speedily unlocking and connecting the data. (Pilsk, S.C., et al. “The Biodiversity Heritage Library: Advancing Metadata Practices in a Collaborative Digital Library” Journal of Library Metadata 10:136-155, 2010 doi: 10.1080/19386389.2010.506400) Connecting the index to the bibliography and the bibliography to library holdings has required many more hands and eyes than lines of script. Staff, interns and volunteers began to attempt to locate standard library records for each title in the bibliography.

**Figure 5.**
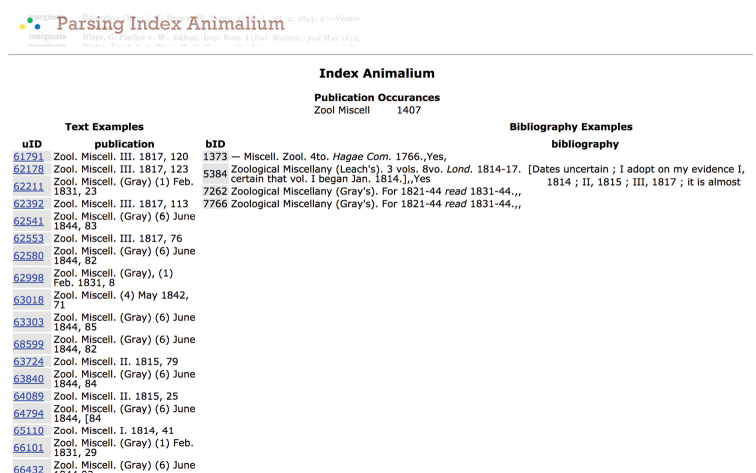
uBio parsing. Microcitation “Zool Miscell” found 1407 times in the *Index Animalium* and potentially matches four entries in Sherborn’s bibliographies.

## IV.

A Microsoft Access database was constructed that contained only the bibliography from *Index Animalium*. Sherborn’s bibliography entries were sorted by the greatest number of associated microcitations. These were searched and the full title, author, date and related identifiers were added to a database. Each line of data from the *Index’s* bibliographies was matched against standard library data using Smithsonian’s online catalogue (SIRIS) and against the OCLC WorldCat catalogue. (Smithsonian Libraries’ online catalog SIRIS is searchable via http://siris-libraries.si.edu.WorldCat is considered to be the largest network of library data. Listing library holdings from around the world, it contains metadata describing these titles following international standards. OCLC’s WorldCat is available for searching http://www.worldcat.org/).

**Figure 6.**
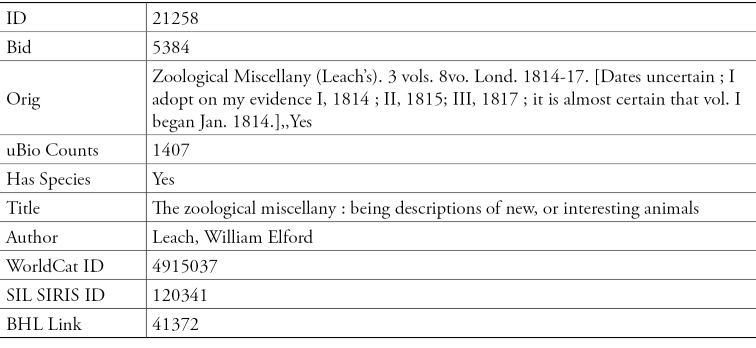
Data from the Smithsonian Libraries database for one line in the *Index Animalium* Bibliography.

**Figure 7.**
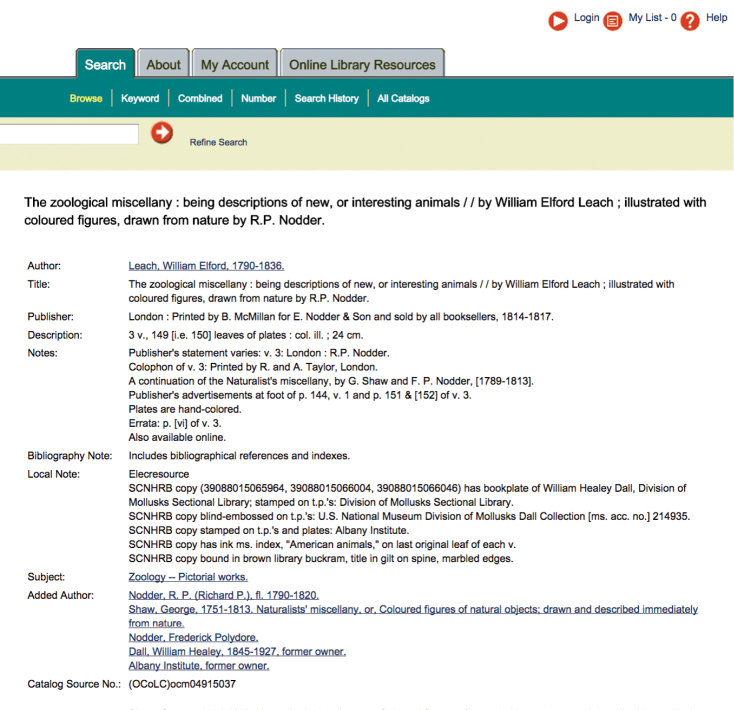
Screen capture of the Smithsonian Institution Online Catalog SIRIS for William Elford Leach’s Zoological Miscellany.

**Figure 8.**
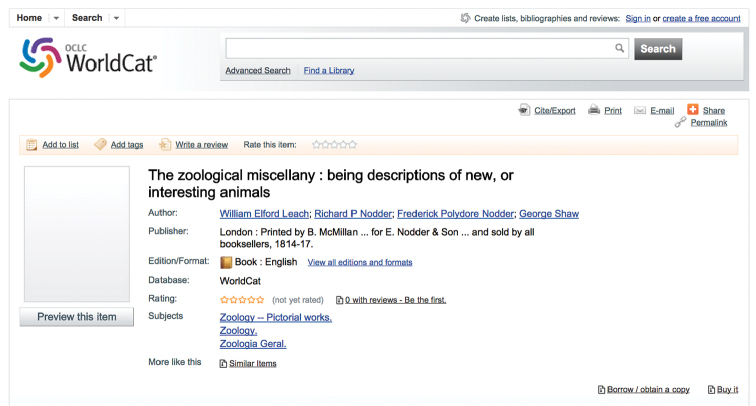
Screen capture from OCLC’s WorldCat for William Elford Leach’s Zoological Miscellany.

## V.

The Smithsonian Libraries’ *Index Animalium* project took a new direction as the Biodiversity Heritage Library (BHL) project began production. A large scale scanning project, BHL’s mission is to digitize legacy natural history literature that is significant in the study and research of biodiversity. BHL is made up of a consortium of international natural history and botanical libraries. Specific funding of the BHL supports the scanning of the literature published before 1923 – titles that Sherborn referenced in his bibliographies. BHL ramped up fairly quickly and began to have full text scans online in 2005. Libraries participating in identifying and scanning the literature stretch across the globe and continue to produce millions of pages of online text ever year. Biodiversity Heritage Library information can be found at http://biodivlib.wikispaces.com/ and the collection is searchable http://www.biodiversitylibrary.org/.

As more and more of the literature becomes available online via the BHL, the *Smithsonian Libraries Index Animalium* project has, once again, shifted in the goal of service to the taxonomic researcher. Instead of getting the researcher to the library shelf for the text, it is becoming more desirable to deliver the fully scanned text to the researcher. Currently the matching of scanned titles is underway with identified titles in the *Index’s* bibliographies. The anticipated result will have the researcher “click and go” from Sherborn’s *Index* online to the BHL scanned text online. Apparently seamless, the connections being made behind the scenes match the online *Index’s* microcitation to the full title record and resolve to the proper title in BHL.

**Figure 9.**
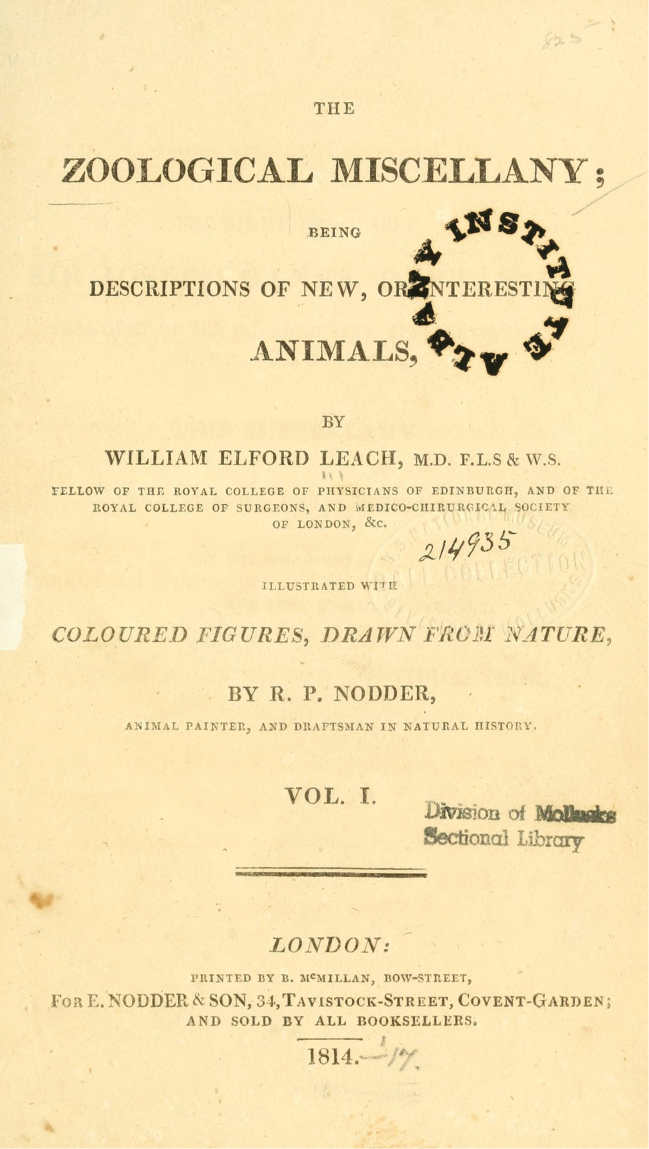
Title page from Biodiversity Heritage Library for William Elford Leach’s Zoological Miscellany.

## VI.

Partnerships forming over the use of Sherborn’s *Index Animalium* distribute the work into more functional pieces to achieve the seamless online research tool. Richard Pyle of the Bishop Museum is applying some reviews to the re-keyed text and providing complete citations for items that have partial data in the Smithsonian database. Another partnership is with Thomson Reuter’s staff working on ION: Index to Organism Names. Thomson Reuters Index to Organism Names (ION) http://www.organismnames.com/ is a free online service to search the names included in *Zoological Record*, a continuously updated database of biological taxonomic research. As Nigel Robinson’s presentation “Sherborn’s *Index Animalium* Integration into ION: Access to All” demonstrated, the parsing of the microcitations and identifying the full text is underway increasing the data in ION and providing the connection needed for the taxonomic researcher. Slides from Nigel Robinson’s presentation are available at http://www.slideshare.net/iczn/4-sherborns-index-animalium-integration-into-ion.

**Figure 10.**
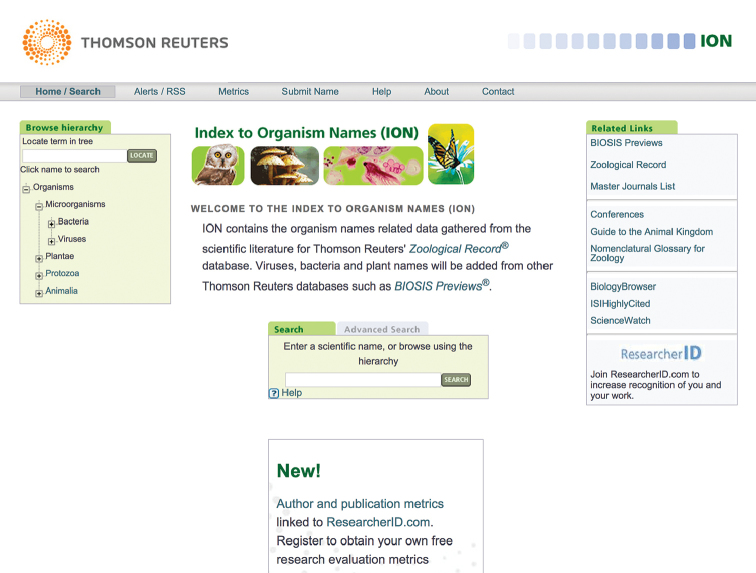
ION home page screen capture.

**Figure 11.**
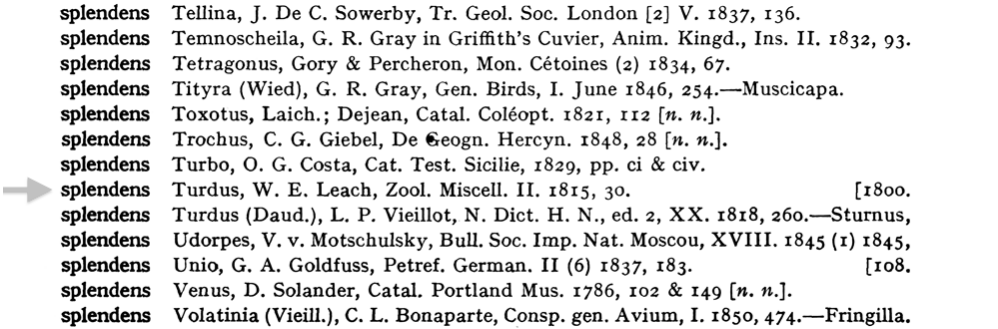
Sherborn citation from page 6101 of the Index Animalium: *Turdus splendens*, W.E. Leach, Zool. Miscell. II. 1815, 30.

**Figure 12.**
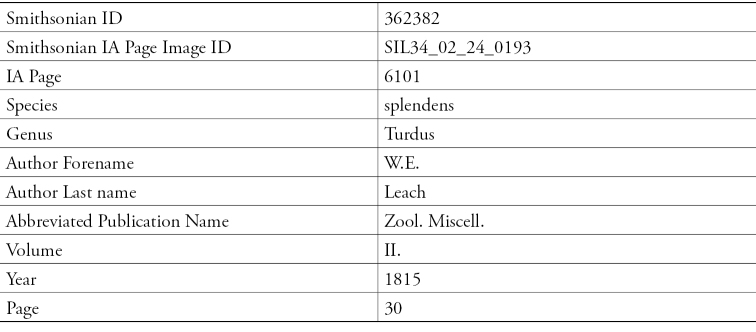
Parsed Data from Smithsonian Libraries Sherborn Database.

The ION team working on *Index Animalium* at Thomson Reuters is looking at supplementing the *Zoological Record* dataset. Sherborn’s data back fills ION with taxonomic names for 1758 to 1864. To achieve the data extraction from *Index Animalium*, Robinson reports that there are challenges in parsing and properly identifying the data elements. The review of the data is needed since Sherborn’s use of commas, brackets and notations all have meanings that need to be carefully interpreted so as to not lose the intention. As the project has progressed, inconsistencies are coming to light that can now be documented. With this detailed look, the ION team is finding re-keying errors, as well as errors made by Sherborn, and the typesetting done based on Sherborn’s initial transcriptions.

ION’s management classification protocol is also being added to the microcitation so that the data can be processed and incorporated into the systems already in place at Thomson Reuter. The species and genus identified in Sherborn are being folded into the overall delivery of data via the ION search. Robinson’s presentation illustrated the parsing with an example of one line of data from Smithsonian Libraries Sherborn Database that teases out the identification of the citation in the Smithsonian database and the various elements.

**Figure 13.**
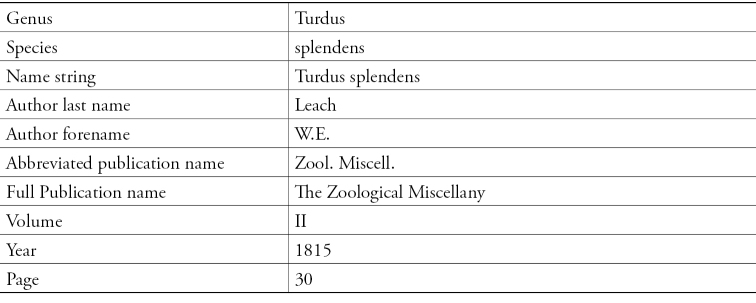
Focusing on the species and genus names, ION is examining the publication abbreviation against the internal Zoological Record data and resolving the microcitations.

**Figure 14.**
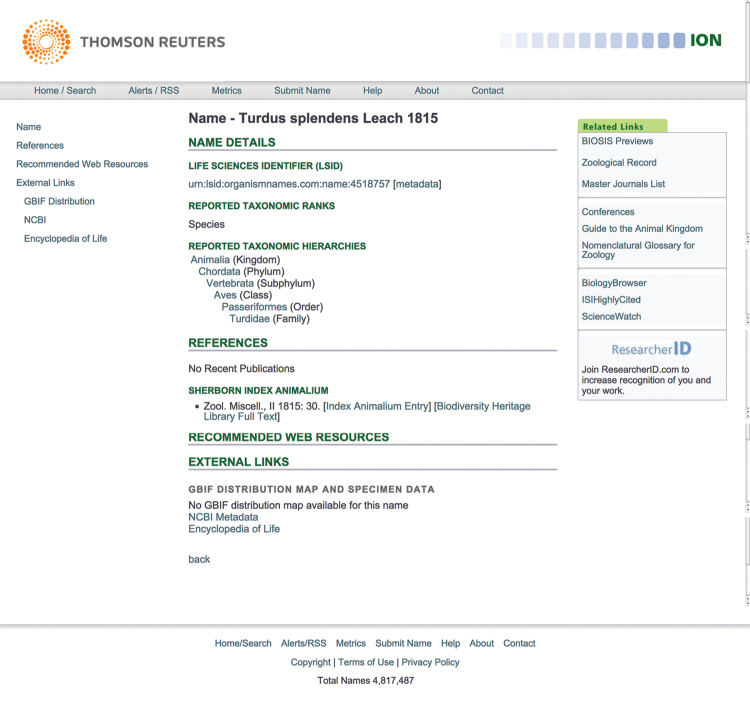
Results of searching in ION for *Turdus splendens*.

**Figure 15.**
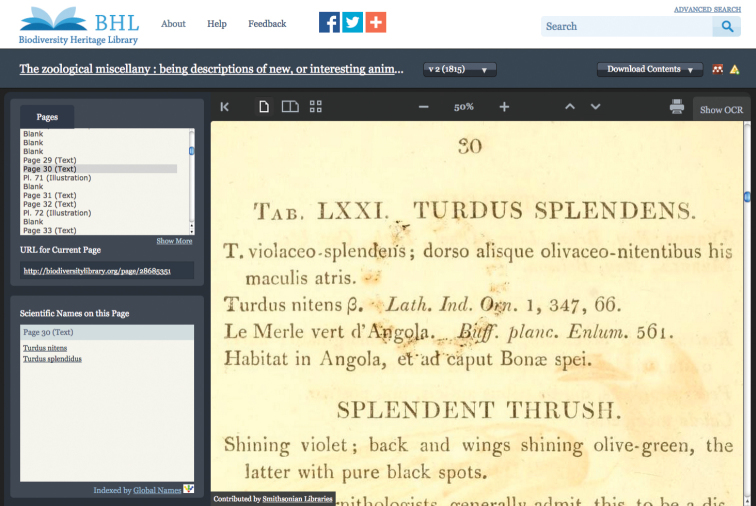
Results of a direct link from ION *Turdus splendens* to the page in the Biodiversity Heritage Library.

(View the page of *Index Animalium* for this reference http://www.sil.si.edu/digitalcollections/indexanimalium/volumes/pagedisplaypage.cfm?filename=SIL34_02_24_0193)

From this breakdown and reconfigurations, ION is able to map data into ION and integrate with the existing ION content to form a nomenclator of names for the literature published from 1758 onwards.

BHL provides stable consistent page identifiers for all titles scanned. In this example *Turdus splendens* page identifier http://biodiversitylibrary.org/page/28685351 is a persistent identifier allowing ION to create a direct link into the Biodiversity Heritage Library. The results are the “click and go” for the user to reach the page of the text Sherborn cites.

## VII.

The challenges of working with legacy taxonomic citations, computer matching algorithms, and making connections have not stopped the attempts to continually improve the reach of Sherborn’s unique and critical data to the researcher. New developments and constant revisiting of the goals has brought us to yet another shift to today’s goal of making Sherborn’s *Index* available and linked to other datasets. The Smithsonian Libraries is exploring a different data structure than a relational database currently in use. Partnering with others in the world of metadata development and information sharing has led to an attempt to allow machine-to-machine communications. The *Index* is being looked at as the data set of the elements it contains. These data points are being examined for possible transformation into RDF mark up and meeting the standards of Linked Open Data. This will allow for broader discovery and access than a stand-alone database. Linked Open Data is primarily aimed at consumption by computer software, but the availability of such data allows the offering of an online research tool geared towards the general population of natural history researchers.

Linked Data is based on the concept of triples or a sentence made up of three parts: subject, predicate, and object. The subject is an identifiable “thing” that can be assigned a unique identifier. The predicate can be considered the “verb” with a controlled vocabulary that has a term defined and assigned a unique identifier. The object is the last “thing” in the triple that subject is connected. A possible triple that would be created from Sherborn’s *Index Animalium* is diagrammed below. In this scheme, each species is presented as an identifier with related microcitation data pointing to the scanned title and page at BHL. The goal of providing the Index as an open data set in the RDF would allow others to reuse, repurpose, and mine the data.

**Figure 16.**
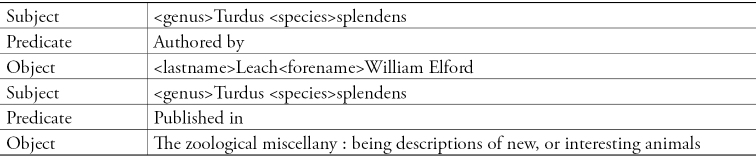
Example of potential triples from Index Animalium’s citation for “splendens Turdus".

The details of creating a complete open linked data set out of *Index Animalium* are still being discussed and explored. Smithsonian Libraries, dedicated to providing data in an open platform, is already beginning to work on providing some Open Linked Data in a new project based off the *Taxonomic Literature: A selective guide to botanical publications and collections with dates, commentaries and types*, 2^nd^ edition. Known by most as TL2, the entire 15 volume set has been scanned and OCRed. The data is currently available for searching and the break down into triples has begun. Smithsonian Libraries TL2 online (http://www.sil.si.edu/digitalcollections/tl-2) allows for reading or searching the entire text of the literature of systematic botany published between 1753 and 1940. Incorporating *Index Animalium*, Smithsonian’s goal is to create a TL3: an online resource containing both botanical and zoological linked open data resource for taxonomic research.

## VIII.

A project that began to simply provide a URL for anyone in the world to read Sherborn’s *Index Animalium* has grown and changed as the fast paced world of knowledge sharing has adapted to the technology available. The *Index* has matured from the pieces of paper of Charles Davies Sherborn’s carefully indicated notes of species citations to a linked data structure. The overarching goal of providing access has been achieved but there is room for it to improve by making the information usable, repurpose-able, and integrated into the researcher’s workflow.
